# Supplementary material for: Rapid turnover of life-cycle-related genes in the brown algae
Source: Genome Biol. 2019 Feb 14;20:35. doi: 10.1186/s13059-019-1630-6 (PMC6374913; doi:10.1186/s13059-019-1630-6)
Supplement: Supplementary file 3 — Protein alignments, corresponding Gblocks html file and CDS sequences for the lineage conserved gametophyte-biased genes. (ZIP 2109 kb) [file 13059_2019_1630_MOESM3_ESM.zip › Con_GA_alignments/Ec-15_002250.aa_ali.fasta-gb.htm]

Ec-15\_002250.aa.fasta.trans.aa\_ali.fasta


## Gblocks 0.91b Results

Processed file: **Ec-15\_002250.aa.fasta.trans.aa\_ali.fasta**  
Number of sequences: **2**  
Alignment assumed to be: **Protein**  
New number of positions: **625** (selected positions are underlined in blue)

```
                         10        20        30        40        50        60
                 =========+=========+=========+=========+=========+=========+
Ec-15_002250     MFDFRGGDYRGGTAPPTFKYPEHSPSDGRPPAVDPHRYCEQDGGAAALPTAPVFRGQRGK
MSTRG.52677.1    ------------------------------------------------------------
                                                                             


                         70        80        90       100       110       120
                 =========+=========+=========+=========+=========+=========+
Ec-15_002250     GGGSTATYKHSHHHSRSGWGKRGPKGRRPYDPMEPKTRPASRQVLSDLMDLNLALKFDKM
MSTRG.52677.1    ------------------------------------------------------------
                                                                             


                        130       140       150       160       170       180
                 =========+=========+=========+=========+=========+=========+
Ec-15_002250     GKQDSTRQPDEQPQQSHAGLTTRTGRRHDGGGARAAEAESSSTLFSDRQARQASRYSSPS
MSTRG.52677.1    -----------------------------XFGAEACRQEGMDMLCH--------------
                                                #############                


                        190       200       210       220       230       240
                 =========+=========+=========+=========+=========+=========+
Ec-15_002250     ERYKRVARARNSAILGASNNEQLDHEDVSTSVKHPAVEKYTRWGDDMQSATPCVPATTTC
MSTRG.52677.1    --------------------DRSDDVGVPTHFDQKAAQKHNGRGDGLHPVPSCV--TSNH
                                        ###############################  ####


                        250       260       270       280       290       300
                 =========+=========+=========+=========+=========+=========+
Ec-15_002250     DIPLMISPSRNSTAHTRNSSQGDQGDEQFNTAPQARDDSRSSAWRSENTNTYRGPEG-GY
MSTRG.52677.1    DFQAALSSSNNHDVHLGKSSQR---KAPHSHNSQRKSLQRKYARRGRRSRDLTLAEHFKA
                 #####################            ############               


                        310       320       330       340       350       360
                 =========+=========+=========+=========+=========+=========+
Ec-15_002250     TPQGFGQGSRSEVDIGVWTEYAEDREQGQHTPSQDTCSQQSEKDQRMWSALKNSLLSGSP
MSTRG.52677.1    SARKSVQVHSTAVEATDWAEQTEDRDQGDISLFHQTCNHQNDKDQRMWSALRTSLLSISG
                       ######################################################


                        370       380       390       400       410       420
                 =========+=========+=========+=========+=========+=========+
Ec-15_002250     RDMEGSRFEFQNGRIDDGGN--------LDGG-TDPRDLEQLDLERSAMDRTMRPHRVAS
MSTRG.52677.1    EDTDPTIVGLRIGSIEDGRSSDGGSACNRSSRIVVRRDMEQLHLERSAMDRTIRPHRIAS
                 ##                                  ########################


                        430       440       450       460       470       480
                 =========+=========+=========+=========+=========+=========+
Ec-15_002250     ARLLEKTQNSSVQAKVKQVRTFSASATRPRTRQGESASSVWAGAGGILQCTGVRAELVSR
MSTRG.52677.1    ARALEASQDSPLQTKVKQPRTFSASARRSRTRKNDAAS--------TLRCTGIRAELVSR
                 ######################################         #############


                        490       500       510       520       530       540
                 =========+=========+=========+=========+=========+=========+
Ec-15_002250     NGDGQSSYN---REAASGHSTFAGEAVNRRRESVHQQARKKELGCSRWVTNDEGVANFLQ
MSTRG.52677.1    SGDAQSGFSNETPDTADGADDSAIIGSNCRQKGAQHVARKTDMRRFQWVTNDEGVANFLQ
                 ######         #############################################


                        550       560       570       580       590       600
                 =========+=========+=========+=========+=========+=========+
Ec-15_002250     RPPSRQKEAFPTHLADVVPYAAFQVEVPMTDEEAKQLIEATRKTAVEQTRPPSRHKPPEQ
MSTRG.52677.1    RPPSRQKEAFPTHLADVVPYPAFQVEAPTTDERAKQLAEATRKNAVEQSRPPSRHKPPGQ
                 ############################################################


                        610       620       630       640       650       660
                 =========+=========+=========+=========+=========+=========+
Ec-15_002250     SLFLEVPETRRQIPPALSPSRRMLPHGTESCSALSPGVFSIDVPEAEASPSARDALGTIF
MSTRG.52677.1    SLFLEVPEPRRQVQPAFAFPRGAPPTGAESCDGTAH---SVDSADVEASSTTRDALEVIF
                 ###############################        #####################


                        670       680       690       700       710       720
                 =========+=========+=========+=========+=========+=========+
Ec-15_002250     ALDRRHDNPDRDGFSVLQVQGPCPFRIEVTNSFVQAQPP-KAPVDRETVVAPDARKAAKY
MSTRG.52677.1    ALDRRHDNPDRDGFSVLQVQGPCPFRIEVTNSLMQPQPPTVAPNGREAVDEANPRKVAKH
                 #######################################  ###################


                        730       740       750       760       770       780
                 =========+=========+=========+=========+=========+=========+
Ec-15_002250     RPPDPSCGLFGPMACDSGENASAGKQRAPINTTPNRAGLAIGSPVPARSHTAGKNCGMSF
MSTRG.52677.1    CTPDPSCGLFGSMSYDTCGRTGTEKQRAPINTTPTLAASAIGSSAPTRGDIVGE---SSY
                 #####################################################     ##


                        790       800       810       820       830       840
                 =========+=========+=========+=========+=========+=========+
Ec-15_002250     RLLGSRSTGGITSTT----APCSIRGRDNSSAGAIPSPSPRTGNVNHEPTDEGRPGTPEV
MSTRG.52677.1    RLIGSRSTGGIALRGANIPGTCPVRSEDNGIGGGLSMPPPREGAENRELTAGG--AVITE
                 ###########          ################################       


                        850       860       870       880       890       900
                 =========+=========+=========+=========+=========+=========+
Ec-15_002250     GEFSGLVDGAVENSAGRRANAAAGGNSPSAGRRDSSGLVVSTAASGQNVADWSLQSCDDA
MSTRG.52677.1    AEDRRQGERFAEDCVERGIKGTEGGNVLSIGRGNNSGLFVNTGAPGPHVSDWSLQSCDDV
                            #################################################


                        910       920       930       940       950
                 =========+=========+=========+=========+=========+===
Ec-15_002250     DDGAFLD-QVPTLGEADGTHPSRLETTLGQDFLRLFAPSSSHNNGSGLGELHS
MSTRG.52677.1    DDGAFFDGPALVLSESGGTKHSHLETTLGQDFLRLFAPSSGRENTSGTREVP-
                 #######     ######################################
```

```
Parameters used
Minimum Number Of Sequences For A Conserved Position: 2
Minimum Number Of Sequences For A Flanking Position: 2
Maximum Number Of Contiguous Nonconserved Positions: 8
Minimum Length Of A Block: 10
Allowed Gap Positions: None
Use Similarity Matrices: Yes
```

```
Flank positions of the 14 selected block(s)
Flanks: [152  164]  [204  234]  [237  261]  [274  285]  [307  362]  [397  458]  [468  486]  [496  631]  [640  699]  [702  773]  [779  791]  [802  833]  [852  907]  [913  950]  

New number of positions in Ec-15_002250.aa.fasta.trans.aa_ali.fasta-gb:  625  (65% of the original 953 positions)
```
